# The Cytotoxic Necrotizing Factor of *Yersinia pseudotuberculosis* (CNFy) is Carried on Extracellular Membrane Vesicles to Host Cells

**DOI:** 10.1038/s41598-018-32530-y

**Published:** 2018-09-21

**Authors:** Ajay K. Monnappa, Wasimul Bari, Jeong Kon Seo, Robert J. Mitchell

**Affiliations:** 1 School of Life Sciences, Department of Biological Sciences, Ulsan National Institute of Science and Technology, Ulsan, South Korea; 2 UNIST Central Research Facilities, Ulsan National Institute of Science and Technology, Ulsan, South Korea

## Abstract

In this study we show *Yersinia pseudotuberculosis* secretes membrane vesicles (MVs) that contain different proteins and virulence factors depending on the strain. Although MVs from *Y. pseudotuberculosis* YPIII and ATCC 29833 had many proteins in common (68.8% of all the proteins identified), those located in the outer membrane fraction differed significantly. For instance, the MVs from *Y. pseudotuberculosis* YPIII harbored numerous *Yersinia* outer proteins (Yops) while they were absent in the ATCC 29833 MVs. Another virulence factor found solely in the YPIII MVs was the cytotoxic necrotizing factor (CNFy), a toxin that leads to multinucleation of host cells. The ability of YPIII MVs to transport this toxin and its activity to host cells was verified using HeLa cells, which responded in a dose-dependent manner; nearly 70% of the culture was multinucleated after addition of 5 µg/ml of the purified YPIII MVs. In contrast, less than 10% were multinucleated when the ATCC 29833 MVs were added. Semi-quantification of CNFy within the YPIII MVs found this toxin is present at concentrations of 5 ~ 10 ng per µg of total MV protein, a concentration that accounts for the cellular responses seen.

## Introduction

*Yersinia pseudotuberculosis* is a Gram-negative pathogen believed to be a direct evolutionary ancestor of *Yersinia pestis*, the causative agent of bubonic plague^1,2^. As a pathogen, *Y. pseudotuberculosis* causes disease and zoonotic infections in both humans and a variety of animals, where it is often transmitted via the fecal-oral route^3,4^. In humans, *Y. pseudotuberculosis* is associated with several medical conditions, including the gastrointestinal disease yersiniosis^5^, mesenteric lymphadenitis^6^, fatal septicemia^7^ and, at times, may lead to post-infectious reactive arthritis^8^.

This pathogen has evolved an exquisite method for delivering virulence factors and effectors into cells of the host, where they inhibit signaling cascades and block the cell’s response to infection^8^. One way it achieves this is through its type-three secretion system (TTSS)^9^, which plays a crucial role in effector translocation into host cells. This pathogen also expresses the invasin (INV), attachment invasion locus (AilA) and adhesion (YadA) proteins^10,11^, all of which mediate its tight adhesion to and entry into mammalian cells.

This is not to say that all strains of *Y. pseudotuberculosis* are created equal. For instance, some strains harbor a large (typically around 70-kb) virulence plasmid that encodes for the *Yersinia* outer proteins (Yops)^12,13^. This is true for *Y. pseudotuberculosis* YPIII, while *Y. pseudotuberculosis* ATCC 29833 does not appear to have this plasmid. Upon intimate contact with a target host cell, the Yops are delivered into the host cell via its Type III Secretion System (T3SS)^14^. In addition, not all strains of *Y. pseudotuberculosis* produce a functional cytotoxic necrotizing factor (CNFy)^15^. This toxin activates the host Rho GTPase^16,17^ by deamidating the Gln63 residue^18^ and contributes to the activity of the Yop proteins^16,19^. Although CNFy and its virulent mechanisms are well known, the means by which this protein is introduced into the host cells has thus far remained unclear. However, the study by Lockman *et al*. (2002) found cell-free supernatants from *Y. pseudotuberculosis* YPIII cultures retained the ability to induce multinucleation^15^, suggesting at the time that CNFy was secreted extracellularly.

Recent research, however, has established many bacterial strains, including both Gram-negative and Gram-positive, secrete membrane vesicles (MVs)^20,21^ to be used in a variety of functions, including in defense^22^, the horizontal transfer of genes^23,24^ and in pathogenesis^25^. This is also true for *Y. pestis*, where the MVs produced were found to contain a number of virulence factors that may contribute to this disease^26^. We report here that its relative, *Y. pseudotuberculosis*, also secretes MVs and that this pathogen uses them to mediate the transfer of the CNFy toxin to host cells.

## Results and Discussion

**Figure 1:**
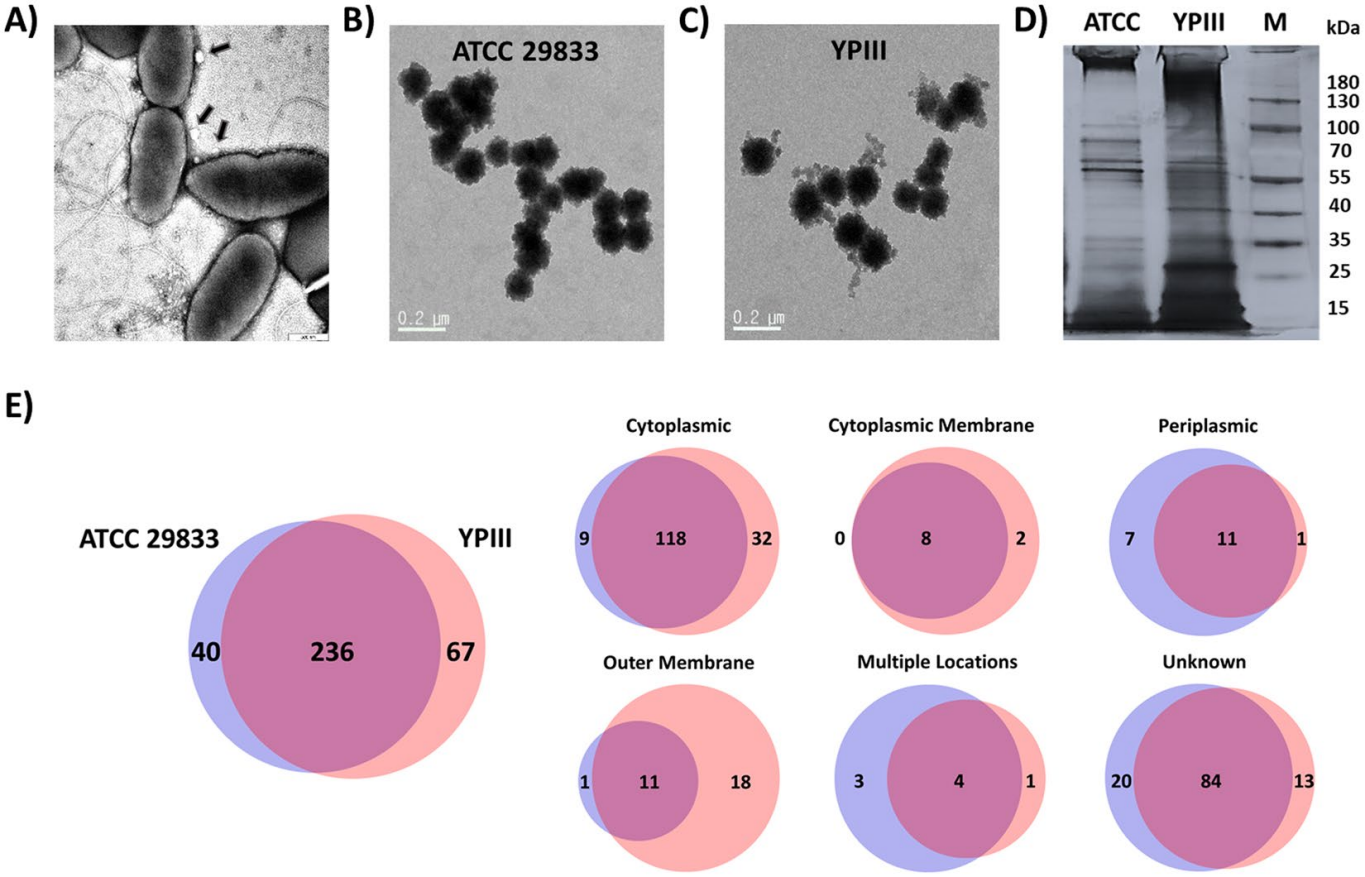
*Y. pseudotuberculosis* secretes MVs and the proteomes differ based on the strain. (**A**) TEM image showing MVs (arrows) budding off of *Y. pseudotuberculosis*. (**B**,**C**) TEM images of MVs purified from cultures of *Y. pseudotuberculosis* ATCC 29833 (**B**) and YPIII (**C**). (**D**,**E**) The proteomes of the two MVs differed based upon SDS-PAGE analyses (**D**) and LC-MS-MS (**E**). (**D**) Distinct bands can be seen in the respective MVs, hinting at differences in their proteomes. The full gel is shown in Fig. S1. (**E**) The LC-MS-MS analyses found a high degree of similarity in the MV proteomes but also some significant difference, particularly in the outer membrane fraction.

Figure 1A shows *Y. pseudotuberculosis* ATCC 29833 secretes MVs, which were visible as membrane blebs on the surface of the cells, while Fig. 1B shows the MVs after they were purified. We also purified MVs from cultures of *Y. pseudotuberculosis* YPIII (Fig. 1C), a better characterized strain of this pathogen^15,27^. Although the diameters of both MVs were similar, ranging from around 70 to 150 nm, their proteomes were quite distinct from one another, as shown in Fig. 1D,E. The proteins present within both MV preparations were subsequently identified using tandem mass spectrometry and classified according to their localization within the cell using PSORTB v. 3.0^28^. In total, the *Y. pseudotuberculosis* ATCC 29833 MVs contained 276 proteins, while 303 proteins were identified within the *Y. pseudotuberculosis* YPIII MVs (Fig. 1E). For both preparations, approximately half of the proteins were cytoplasmic (Fig. 1E). In fact, the top twenty cytoplasmic proteins, as defined by the number of hits, were found in both MV preparations (Table S1). The presence of cytoplasmic proteins in Gram negative MVs has been documented in several previous studies^26,29,30^. While the cytoplasmic protein content within *Salmonella enterica* and *E. coli* DH5α MVs was around 40%^29,30^, with *Y. pestis* the percentage of cytoplasmic proteins was 58%^26^, a level that affirms the results seen here with *Y. pseudotuberculosis*. The significant presence of cytoplasmic proteins implies these bacteria are generating double membrane vesicles, a type of MV that is produced by other Gram-negative bacterial species, including *Shewanella vesiculosa*^31^, *Vibrio cholerae*^32^ and *Pseudalteromonas marina*^33^, where they are also involved in transporting RNA and DNA. In the *V. cholerae* study, the authors found the larger MVs (>100 nm) were the ones that had double membranes while the smaller MVs tended to be single membrane^32^. As noted above, the average diameter of the MVs in this study were between 70 and 150 nm.

Although a majority of the proteins identified (nearly 70%) were shared by both MVs, clear differences were also seen. For instance, the MVs from YPIII had significantly more outer membrane proteins (OMPs) than those from ATCC 29833 (Figs 1E and S2). The greater number of OMPs in the YPIII MVs is further supported by Table 1, where the top 20 OMPs are listed based upon peptide hits. As listed in this table, all twenty of these proteins were present in the YPIII MVs, including several *Yersinia* outer proteins (Yops) and the CNFy toxin, while the ATCC 29833 MVs carried only four. The complete list of OMPs is provided in Table S1. One potential explanation for the differences between the two MV preparations is the large virulence plasmid that is found within *Y. pseudotuberculosis* YPIII^12^, but has never been reported in strain ATCC 29833. This plasmid encodes for many virulence factors, including YopD, YopE, YopH and YopN, four of the proteins that were found within the YPIII MVs but lacking in the ATCC 29833 MVs (Table 1). During an infection, YopD forms a translocation channel along with YopB within the host cell membrane to inject the other Yop proteins^34^, while YopH, a tyrosine phosphatase, modulates the immune response. It accomplishes this by targeting p130Cas and the focal adhesion kinase (FAK), leading to reduced phagocytosis of *Yersinia* by macrophages^35^, as well as by counteracting T-cell activation^36^. Although Yops are transported into the host cell via the *Y. pseudotuberculosis* T3SS^37^, the proteomic data here suggests they are also be transported via MVs.

**Table 1:** Top 20 outer membrane proteins associated with *Y. pseudotuberculosis* MVs.

No	Gene	Protein	UniProt ID	M.Wt	ATCC	YPIII
1	*cnfY*	Cytotoxic necrotizing factor	B1JQP1_YERPY	115 kDa	−	+
2	*yopD*	YopD family protein	B2KAK2_YERPB	33 kDa	−	+
3	*yopE*	Outer membrane virulence protein YopE	YOPE_YERPS	23 kDa	−	+
4	*yopH*	Protein-tyrosine phosphatase YopH	B2KAF0_YERPB	51 kDa	−	+
5	*yopN*	Type III secretion regulator YopN/LcrE/InvE/MxiC	B2KAL3_YERPB	33 kDa	−	+
6	*hmuR*	TonB-dependent hemin receptor HmuR	A7FNC7_YERP3	74 kDa	−	+
7	*ompA*	Outer membrane protein A OmpA	A7FJS9_YERP3	39 kDa	−	+
8		Porin Gram-negative type	B2K9B2_YERPB	41 kDa	−	+
9		Porin Gram-negative type	B1JQS5_YERPY	40 kDa	−	+
10		Major outer membrane lipoprotein	A7FHJ5_YERP3	8 kDa	−	+
11		Lipoprotein	B1JRX0_YERPY	39 kDa	−	+
12		Peptidoglycan-associated lipoprotein	A7FKQ2_YERP3	18 kDa	−	+
13	*lamB*	Maltoporin LamB	B1JJN5_YERPY	47 kDa	−	+
14		OmpW family protein	B1JKS5_YERPY	23 kDa	−	+
15	*bamA*	Outer membrane protein assembly factor BamA	BAMA_YERP3	88 kDa	−	+
16	*bamB*	Outer membrane protein assembly factor BamB	A7FFZ2_YERP3	42 kDa	−	+
17	*bamC*	Outer membrane protein assembly factor BamC	A7FG50_YERP3	38 kDa	+	+
18	*gdhA*	Glutamate dehydrogenase GdhA	A7FP04_YERP3	48 kDa	+	+
19		Virulence-related outer membrane protein	B1JRY0_YERPY	20 kDa	+	+
20		Lipoprotein	B1JQI8_YERPY	29 kDa	+	+

Another virulence factor that was found only in the YPIII MVs is the cytotoxic necrotizing factor (CNFy, Table 1). This protein is an AB-type, pore-forming toxin produced by *Y. pseudotuberculosis* and pathogenic strains of *E. coli*^38^. Within *Y. pseudotuberculosis* YPIII this toxin is chromosomally encoded by the *cnfy* gene (3,045 bp in length) but is truncated (1.8 kb) in *Y. pseudotuberculosis* ATCC 29833^15^. As a virulence factor, CNFy has been identified within host cells during infection where it contributes to the delivery and activity of the Yops^16,19^, activates Rho GTPase^16,17^ and leads to multinucleation^22^. The mechanism used by *Y. pseudotuberculosis* YPIII to introduce CNFy into host cells, however, has not been established. The cytotoxic necrotizing factor produced by uropathogenic strains of *E. coli* (CNF1), however, is transferred in an active form via MVs^39^. Given the sequence similarity between the CNFy and CNF1 proteins^15^, we hypothesized that the same might be true of CNFy. Although this idea was proposed previously^16^, the authors did not provide any evidence to support this in their study.

**Figure 2:**
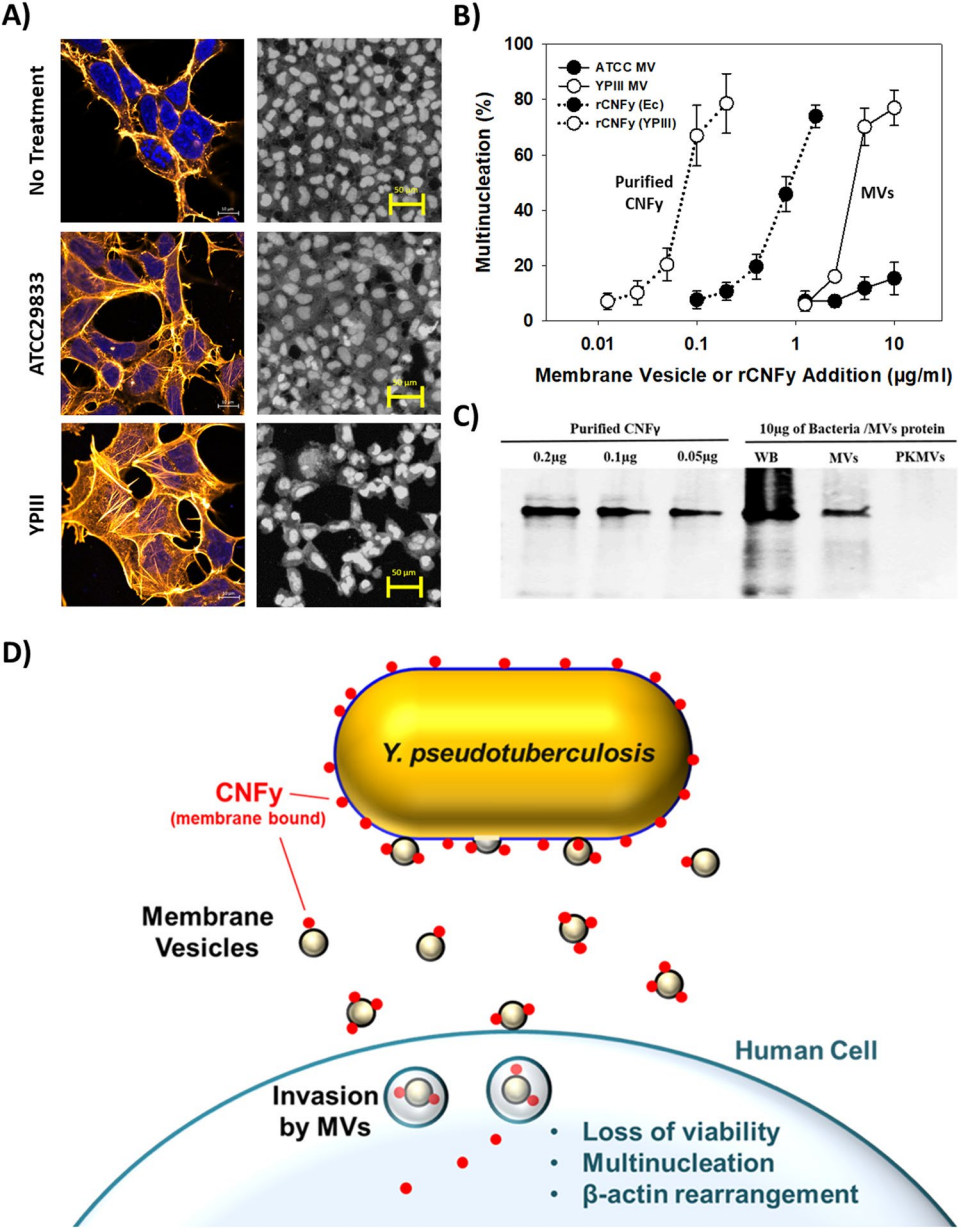
CNFy is transported to host cells via MVs. (**A**) MVs from *Y. pseudotuberculosis* YPIII led to significantly greater responses from the HeLa cell cultures, including actin rearrangement and multinucleation, than those from *Y. pseudotuberculosis* ATCC 29833. (**B**) Percent of cells displaying multinucleation after treatment with the purified MVs or the addition of the his-tagged *Y. pseudotuberculosis* YPIII CNFy. (**C**) Semi-quantification of CNFy within the YPIII MVs using western blotting. All of the samples were probed on the same blot as shown. The results suggest there is approximately 0.05 to 0.1 µg per 10 µg of MVs. Treatment with proteinase K (PKMVs) removed this signal, implying this protein is present on the surface of the MVs. (**D**) Proposed mechanism used by *Y. pseudotuberculosis* YPIII to introduce its CNFy into host cells using MVs.

Although the results above show CNFy was present in the YPIII MVs, it was not clear if the toxin was active or if the MVs transport this toxin to host cells. To evaluate this, we exposed HeLa cells to MVs purified from either *Y. pseudotuberculosis* ATCC 29833 or YPIII cultures. The former was used since the truncated CNFy protein has a reduced activity and does not strongly induce multinucleation^15^. Figure 2A shows the HeLa cultures responses after an exposure to 10 mg/ml of either MV. Whereas the purified YPIII MVs led to both an extensive β-actin rearrangement and multinucleation within the HeLa cells, the ATCC 29833 MVs induced only mild cellular responses by comparison. This is supported by the results in Fig. 2B, which shows the degree of multinucleation increases as the concentration of MVs added increased. Based on these results, a minimum of 2.5 µg of YPIII MVs is needed to elicit a significant response.

Focusing on the cell cultures exposed to 10 µg of MVs, a vast majority of the HeLa cells were multinucleated, proving the MV-bound CNFy is active. These results support the findings of Lockman *et al*. (2002), where filtered cell-free supernatants induced multinucleation of Hep-2 cells^15^. Given their diminutive size, bacterial MVs are not removed by typical filter sterilization methods and, thus, will remain in the media to induce the responses as seen in their study. In contrast, the ATCC 29833 MVs induced only very mild responses from the HeLa cells regardless of the concentration added. This was expected given the CNFy protein expressed by this strain is truncated, making it much less potent and unable to elicit the same level of cellular responses^15^. The YPIII MVs not only led to stronger cellular responses than their ATCC counterparts but were also toxic towards the HeLa cells as evidenced by the Live/Dead stain results in Fig. S3. Moreover, treatment of the YPIII MVs with either heat (95 °C for 10 min) or proteinase K eliminated their activities (Figs S3B and S4), implying the CNFy toxin is located on the surface of the MVs and exposed to the surrounding media.

To prove CNFy is truly associated with YPIII MVs, next we constructed and expressed a C-terminal his-tagged CNFy fusion gene within *Y. pseudotuberculosis* YPIII. When MVs purified from these cultures were probed using anti-his antibodies, CNFy was readily detected (Fig. 2C). A semi-quantitative analysis using purified CNFy suggested its concentration within the MVs was between 0.05 and 0.1 µg per 10 µg of total MV protein. Although the expression of CNFy by *Y. pseudotuberculosis* YPIII was initially reported to be constitutive^15^, a more recent study found this toxin is expressed more strongly when this pathogen is cultured at 37 °C rather than at 25 °C^16,40^. This is also true for the Yops mentioned above, the expression levels increasing between 7.2 and 28.4-fold^40^. As the temperature used in this study was 30 °C, the concentrations of the Yops and CNFy within the MVs will likely be greater under physiological conditions.

When purified CNFy was added to the HeLa cell cultures at the above concentrations, *i.e*., between 0.05 and 0.1 µg/ml, we found they induced similar levels of multinucleation (Figs 2B and S5), implying the activity of the MV-bound CNFy is comparable with that of the free toxin. Agreeing with the results in Figs S3B and S4, treatment of the MVs with proteinase K eliminated any signal in the western blots (Fig. 2C), further proving this toxin is present in the outer membrane of the MVs as listed in Table 1. Additional experiments also found the YPIII MVs associate with, and are likely taken up into, the HeLa cells (Fig. S6), implying they may be used by *Y. pseudotuberculosis* YPIII to introduce this toxin into host cells. This concept is illustrated in Fig. 2D.

In summary, this study shows different strains of *Y. pseudotuberculosis* secrete MVs harboring several different virulence factors. Based upon the genotype, with *Y. pseudotuberculosis* YPIII harboring a large virulence plasmid that is absent in *Y. pseudotuberculosis* ATCC 29833, the MV proteomes differed; the YPIII MVs carried many Yops and CNFy protein, while these were missing in the ATCC 29833 MVs. As such, this is the first study to demonstrate the CNFy toxin of *Y, pseudotuberculosis* can be transported to host cells via MVs and that they can induce cellular responses and death in the absence of whole pathogens.

## Materials and Methods

### Bacterial strains and growth conditions

*Yersinia pseudotuberculosis* ATCC 29833 was obtained from the Korean Agricultural Culture Collection (KACC) (http://genebank.rda.go.kr/eng/uat/uia/actionMain.do)) and *Y. pseudotuberculosis* YPIII was obtained from the Biodefense and Emerging Infections Research Resources Repository (BEI Resources, USA). The bacterial strains were maintained as frozen 20% glycerol stocks at −80 °C. Upon need, they were grown on Lysogeny broth (LB) agar plates (BD; Difco, USA) at 30 °C overnight. From this, a single colony was inoculated into 5 ml of LB broth and grown overnight at 30 °C with agitation (250 rpm).

### Human cell cultures

The HeLa cells, which we purchased from the American Type Culture Collection (ATCC; www.atcc.org) were cultured in T75 flasks in Dulbecco’s Modified Eagles Medium (MEM, 1:1 with sodium bicarbonate (Life Technologies, USA)) supplemented with 10% heat inactivated fetal bovine serum and 100 μg/ml Normocin (Invivogen, USA).

### Purification of the MVs

MVs were purified as mentioned previously^26^, with slight modification. Briefly, the 500 ml bacterial cultures were grown in LB medium at 30 °C and 250 rpm in 2-liter flasks for 24 h (OD600 of ∼2.3). The bacterial cells and debris were removed by low-speed centrifugation (5,000 × g, 30 min) and the medium was sterilized by passing it through a 0.2 μm filter (Millipore, USA). The cell-free medium was concentrated to ~5 ml using a 100-kDa MWCO Amicon filter (USA). The concentrated MVs were pelleted by ultracentrifugation (40000 rpm, 2 h, 4 °C) and suspended in 25 mM HEPES (pH 7.5). They were then mixed with OptiPrep (Sigma Aldrich, USA) to generate a 40% (vol/vol) OptiPrep solution in a total volume of 2 ml. The samples were loaded into a 13.2-ml ultracentrifuge tube, and lower-concentration OptiPrep solutions were layered on top (2 ml [40%], 2 ml [30%], 2 ml [25%], 2 ml [20%], 1 ml [15%] and finally 0.5 ml [0%]). The samples were centrifuged (100,000 × g, 16 h, 4 °C) in a swinging-bucket rotor, after which 1 ml fractions were collected sequentially from the top of the tube. The protein content in each fraction was examined by 10% Tris-glycine SDS-PAGE gels (Biorad, USA). Adjacent fractions with similar protein profiles were combined, diluted into 25 mM HEPES (pH 7.5) and recovered via ultracentrifugation (100,000 × g, 2 h, 4 °C). The recovered pellets were then resuspended in 25 mM HEPES (pH 7.5) and the MV concentration determined using the Bradford assay.

### TEM analysis of the bacteria and MVs

For the TEM images, 2 µl of purified MVs or bacterial culture were placed on carbon-coated grids (Polysciences, Inc., USA). The samples were treated with 2.5% glutaraldehyde for 1 h, washed sequentially with increasing amounts of ethanol (40%, 50% 60% 80% 90% and 100%) and finally in absolute acetone. The samples were then negatively stained with 0.1% uranyl acetate and examined under a transmission electron microscope (JEOL 1200 EX, USA).

### Proteomic analysis of *Yersinia pseudotuberculosis* MVs

To identify the proteins within the MVs, triplicate samples for both *Y, pseudotuberculosis* ATCC 29833 and YPIII cultures were subjected to in-gel digestion. For this 10 μg of the MVs (based on the Bradford assay) were separated on 10% Tris-glycine SDS-PAGE gels (Biorad, USA). After staining with coomassie blue, each lane was cut into six pieces and the proteins in each were subjected to in-gel tryptic digestion as described by Shevchenko and co-workers^41^. The resulting tryptic peptides were analyzed by LC-MS/MS. All the mass analyses were performed on an LTQ-Orbitrap (Thermo, Bremen, Germany) equipped with a nanoelectrospray ion source. To separate the peptide mixture, we used a C18 reverse phase HPLC column (150 mm × 75 um i.d.) using an acetonitrile/0.1% formic acid gradient from 10 to 24% for 90 min at a flow rate of 300 nl/min.

For the MS/MS analysis, the precursor ion scan MS spectra (m/z 400–2000) were acquired in the Orbitrap at a resolution of 60,000 at m/z 400 with an internal lock mass. The 20 most intensive ions were isolated and fragmented in the linear ion trap by collisionally induced dissociation (CID). All the MS/MS samples were analyzed using Sequest (Thermo Fisher Scientific, San Jose, CA, USA; version 1.4.1.14) and X! Tandem (The GPM, thegpm.org; version CYCLONE (2010.12.01.1)). Sequest and X! Tandem were set up to search the *Y. pseudotuberculosis* protein sequence database (9823 entries, UniProt (http://www.uniprot.org/)) assuming digestion by trypsin. Sequest and X! Tandem were searched with a fragment ion mass tolerance of 0.60 Da and a parent ion tolerance of 10.0 ppm.

Scaffold (version Scaffold_4.4.3, Proteome Software Inc., Portland,) was used to validate the MS/MS-based peptide and protein identifications. Protein identifications were accepted only if they could be established at greater than 99.0% probability to achieve a false detection rate of less than 1.0% and contained at least two identified peptides. Protein probabilities were assigned by the Protein Prophet algorithm. Proteins that contained similar peptides and could not be differentiated based on MS/MS analysis alone were grouped to satisfy the principles of parsimony. The proteins were annotated with their gene ontology terms, which were obtained from the NCBI database (downloaded Aug 1, 2016). Localization of the identified proteins were manually determined using PSORTB v. 3.0 bacterial protein subcellular localization prediction program^28^.

### Cloning and expression of His-tagged CNFy

The gene encoding for the CNFy toxin within the genome of *Y. pseudotuberculosis* YPIII was amplified by PCR using primers CNF-F (5′-TATTCTAGATTTTACATGTAATAATAAATAGCAG-3′) and CNF-R (5′-ATACTCGAGAAAGTCTTTTTGTAAAACATTAAAC-3′). These primers introduced restriction sites for *Xba*1 and *Xho*1 (underlined regions in the primers, respectively). The resulting PCR product was digested using these enzymes, ligated into pET-31b+ to generate a 6X his-Tag fusion and transformed into *E. coli* DH5α. After confirming the insert was correct, this plasmid (pET31B + CNFy) was purified and transformed into *Y. pseudotuberculosis* YPIII.

### Purification of CNFy

*Y. pseudotuberculosis* YPIII harboring pET31B + CNFy was grown in LB medium with 100 µg/ml ampicillin added. When the culture reached an OD of 1.0, the cells were collected by centrifugation at 5,752 × g (15 min), washed with PBS and then suspended in lysis buffer (50 mM Tris-HCl pH 7.5; 200 mM NaCl) supplemented with lysozyme (1 mg/ml). This suspension was kept on ice for 30 min before sonication (20% amplitude for 30 sec pulses). After sonication, the cell debris was removed by centrifugation at 5,752 × g for 30 min at 4 °C. The recombinant CNFy (rCNFy) was then purified by affinity chromatography using Ni-NTA agarose beads (Qiagen, USA) according to the manufacturer’s suggested protocol. The purified protein was then quantified using the Bradford assay. To confirm that the protein was pure, a sample was separated using a 10% SDS PAGE gel and visualized after staining with colloidal coomassie (Fig. S7). Expression of rCNFy within *Y. pseudotuberculosis* YPIII was also confirmed by western blotting with the Penta·His HRP Conjugate Kit from Qiagen (USA) (Fig. 2C).

### Semi-quantitative analysis of CNFy in MVs using western blotting

Samples containing 10 μg of protein from whole cells or MVs from *Yersinia pseudotuberculosis* YPIII pET31b-CNFy were separated on 4–20% Tris-glycine SDS-PAGE gels (Biorad, USA). Samples of purified rCNFy (0.2 µg, 0.1 µg and 0.05 µg) was also run on the same gel. Subsequently, the proteins were electro-blotted onto a polyvinylidene difluoride (PVDF) membrane (GE health care, USA) using a semi-dry transblotter (Biorad, USA). The membrane was then incubated for 2 h in a blocking solution (5% wt/vol skim milk (Difco, USA) in Tris-buffered saline (TBS; 20 mM Tris-HCl buffer (pH 7.6) containing 137 mM NaCl). After three sequential washes with TBS, the membrane was probed with a Penta·His-HRP conjugate (Qiagen, USA) for 2 h using 5% skim milk in TBS. The blot was then washed three times with TBST (TBS containing 0.05% (vol/vol) Tween-20). The signals were then developed using the Supersignal West Femto kit (Thermo scientific, USA) according to the manufacturer’s suggested protocol, and captured using the LAS 200 System (GE Healthcare, USA).

### Immunostaining and confocal microscopy

To inspect the HeLa cells for any morphological changes induced after being exposed to the MVs, confluent monolayers were prepared on Labtek™ II chambered cover glasses (Nunc, Germany). Before treatment, *i.e*., addition of the MVs, the HeLa cells were serum starved for at least 20 h. The cells were then treated with the purified MVs for 12 h, subsequently washed with DPBS and fixed with 3.7% paraformaldehyde (in PBS) for 20 min at room temperature. After fixing the cells, they were washed with DPBS and permeabilized with 0.1% Triton X-100 (Sigma Aldrich, USA) in DPBS for 5 min. The actin cytoskeleton was then stained with a phalloidin-rhodamine solution (0.5 μ g/ml in DPBS; Invitrogen, USA) for 30 min at room temperature. After washing the cells once more with DPBS, the cellular DNA was counter-stained with DAPI (1 μg/ml in PBS) (Life Technologies, USA) for 5 min at room temperature. These cells were then visualized using a laser confocal microscope LSM 780 (Carl Zeiss, Germany) and the captured images processed using Zen 2009 software.

### Quantifying multinucleation

To determine the degree of multinucleation, HeLa cells were seeded into 96 well tissue culture plates (10,000 cells/well) (Corning, USA) and incubated for 24 h with 5% CO_2_. The cells were then incubated with the different concentrations of either the MVs or purified CNFy for 48 h under 5% CO_2_. After this incubation step, the cells were washed with DPBS (Sigma-Aldrich, USA) and fixed with 3.7% paraformaldehyde (in PBS) for 10 min at room temperature. After washing the fixed cells three times with DPBS, they were counterstained with DAPI (1 μg/ml in PBS) (Life Technologies, USA) for 5 min at room temperature. The cells were then visualized using a laser confocal microscope LSM 780 (Carl Zeiss, Germany) and the captured images processed using the Zen 2009 software. The number of multinucleated cells from confocal images were visually counted and used to calculate the percentage.

### Visualization of MVs associated with the HeLa cells

MVs were purified from cultures of *Y. pseudotuberculosis* ATCC 29833 and YPIII as described above and then labeled with Octadecyl Rhodamine B Chloride (R18) (0.5 μg/ml in DPBS; Invitrogen, USA) for 20 min. After staining, any unbound dye was removed by ultracentrifugation at 40,000 rpm for 2 h. Monolayers of the HeLa cells were prepared in 8 well Nunc Lab-Tek II chambered cover glass plates (Thermo Scientific, USA) by seeding 2 × 10^4^ HeLa cells/well and incubating them in a CO_2_ incubator at 37 °C for 24 h. To initiate the experiment, 0.5 ml of the rhodamine BR-18 stained MVs (10 µg/ml in MEM) were added to the wells and the plate was incubated for 2 h at 37 °C before the HeLa cells were washed with DPBS. The cells were then fixed with 3.7% paraformaldehyde (in PBS) for 20 min at room temperature, washed with PBS and permeabilized with a solution of 0.1% Triton X-100 (Sigma Aldrich, USA) in PBS for 5 min.

To visualize the HeLa cells, the actin cytoskeleton was stained using Alexa Fluor 488 phalloidin (0.5 μg/ml in DPBS; Thermo Scientific, USA) for 30 min at room temperature. After washing the cells with DPBS, the cellular DNA was counter-stained with DAPI (1 μ g/ml in PBS; Thermo Scientific, USA) for 5 min at room temperature. The cells and MVs were then visualized using a laser confocal microscope (LSM 780; Carl-Zeiss, Germany). The images were processed using Zen software (Carl-Zeiss, Germany).

### Cell viability assays

The impact of the MVs on the viability of the cultured HeLa cells was determined using the MTT assay according to the manufacturer’s suggested protocol. Briefly, 1 × 10^4^ cells were seeded into the wells of a 96-well plate and grown for 24 h. The cells were then exposed to the MVs from *Y. pseudotuberculosis* ATCC 29833 or YPIII by replacing the media within the wells with fresh media containing MVs. The cells were grown for an additional 24 h in the presence of the MVs. Afterwards, 5 μg/ml of 3-(4,5-dimethyl-2-thiazolyl)-2,5-diphenyl-2H-tetrazoliumbromide (MTT reagent, Life Technologies, USA) was added to each well and the plates were incubated at for an additional 4 h at 37 °C in the dark. After thoroughly removing the media, 400 μl of DMSO was added to each well and the plate was incubated with shaking (150 rpm) at room temperature for 15 min to allow the color to develop. The OD was measured at 550 nm and used as a proxy for the HeLa cell viability.

### Live/Dead imaging

The live and dead cells were labeled using two dyes, calcein AM and ethidium homodimer-1 (EthD-1), respectively. For this, the HeLa cell cultures were prepared by seeding 1 × 10^4^ cells/well in 96-well plates and grown as above. After 24 h, the cells were treated with either media alone or media containing MVs purified from either *Y. pseudotuberculosis* ATCC 29833 or YPIII cultures. After the cells were grown for an additional 24 h in the presence of the MVs, they were washed gently with 1X Dulbecco’s phosphate-buffered saline (DPBS) at room temperature. To each well, a 100 μl mixture of the Live/Dead Assay reagents (containing 1 μM calcein AM and 2 μM EthD-1 (Life Technologies, USA)) was added. After a 10 min incubation at room temperature, the cells were observed using confocal microscopy (LSM 700; Carl Zeiss, Germany).

### Statistical analysis

The data is expressed as the mean (average) of at least three independent replicates with the standard deviations shown as the error bars. An unpaired Student’s t-test was used to analyze the data. P-values of <0.05 were considered statistically significant.

## Electronic supplementary material

Supplemental Figures
