# Supplementary material for: The Cytotoxic Necrotizing Factor of Yersinia pseudotuberculosis (CNFy) is Carried on Extracellular Membrane Vesicles to Host Cells
Source: Sci Rep. 2018 Sep 21;8:14186. doi: 10.1038/s41598-018-32530-y (PMC6155089; doi:10.1038/s41598-018-32530-y)
Supplement: Supplementary file 1 — Supplemental Figures [file 41598_2018_32530_MOESM1_ESM.pdf]

Supplemental Figures

for

The Cytotoxic Necrotizing Factor of *Yersinia pseudotuberculosis* (CNFy) is Carried on Extracellular Membrane Vesicles to Host Cells

Ajay K. Monnappa [1,*], Wasimul Bari [1], Jeong Kon Seo [2] and Robert J. Mitchell [1,*]

[1] School of Life Sciences, Department of Biological Sciences

[2] UNIST Central Research Facilities

Ulsan National Institute of Science and Technology

Corresponding Author Information:

E-mail: AKM – ajaykm@unist.ac.kr; RJM - esgott@unist.ac.kr

Tel: +82-52-217-2513

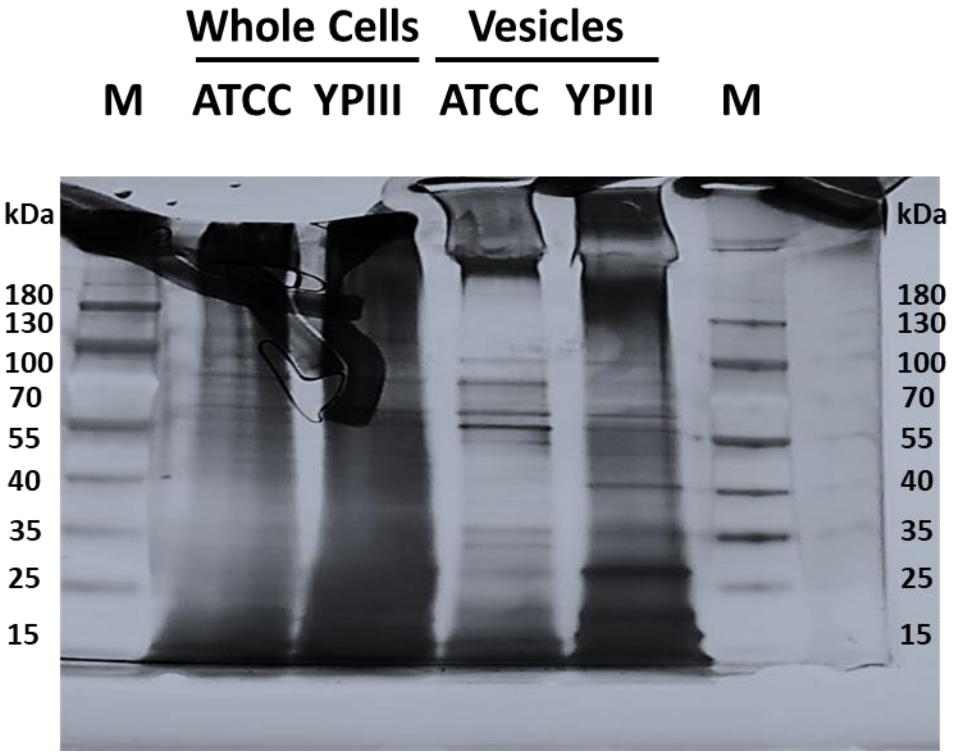

Figure S1. **Image of the SDS-PAGE gel shown in Figure 1D, including the lanes where whole cell extracts were separated. In both cases (whole cells and purified MVs) 10 µg of total protein (based on the Bradford assay) was loaded into the wells. M – Pre-stained protein markers.**

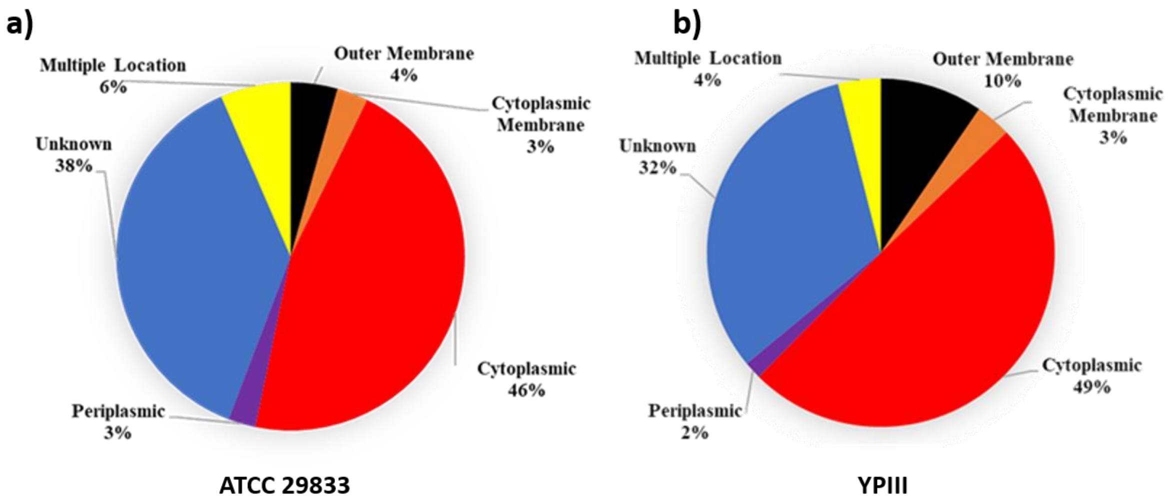

Figure S2. **Subcellular localization of proteins identified in the MVs from *Y. pseudotuberculosis* ATCC 29833 (a) and YPIII (b).**

A)

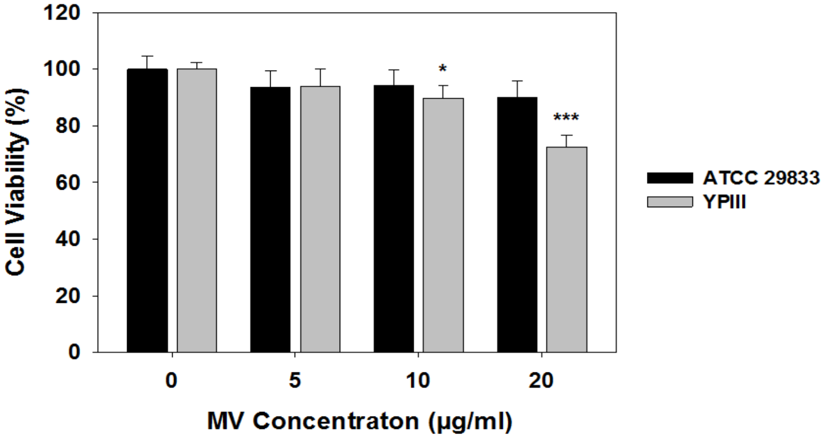

B)

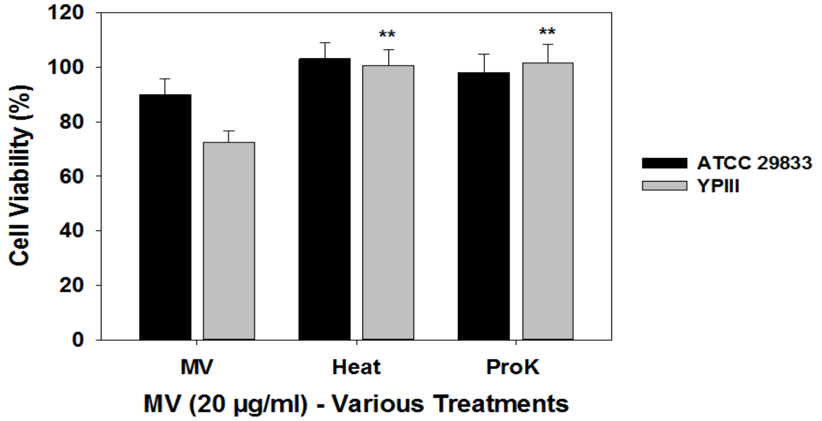

Figure S3. Toxicity of the *Y. pseudotuberculosis* MVs towards HeLa cells using the MTT assay. A) The MVs from *Y. pseudotuberculosis* YPIII were more toxic than those from ATCC 29833, with the addition of 10 and 20 µg/ml leading to significant losses in the HeLa cell viabilities. B) Treatment of the MVs with heat (95ºC for 10 min) or proteinase K completely eradicated their toxicity, significantly improving the HeLa cell viabilities. * = $p < 0.05$; ** = $p < 0.01$; *** = $p < 0.001$

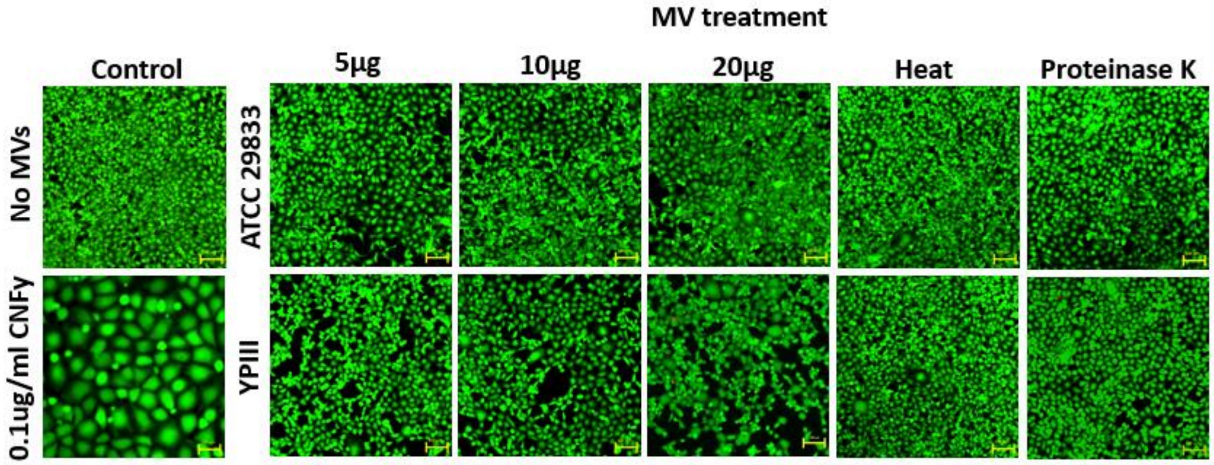

Figure S4. Toxicity of the *Y. pseudotuberculosis* MVs towards HeLa cells using Live/Dead staining. The HeLa cells were treated with different concentrations of the MVs or with 0.1 µg/ml of CNFy purified from *Y. pseudotuberculosis* YPIII. Likewise, 20 µg/ml of MVs treated with heat (95ºC for 10 min) or proteinase K were also tested. After 24 hrs, the HeLa cells were stained with Live/Dead stains (1 µM calcein AM and 2 µM EthD-1) before being imaged using confocal microscopy. The results show the *Y. pseudotuberculosis* YPIII MVs are more toxic and virulent than those from *Y. pseudotuberculosis* ATCC 29833. In addition, treatment of the MVs with either heat or proteinase K completely eradicated their toxicity and virulence. Scale bar-100µm

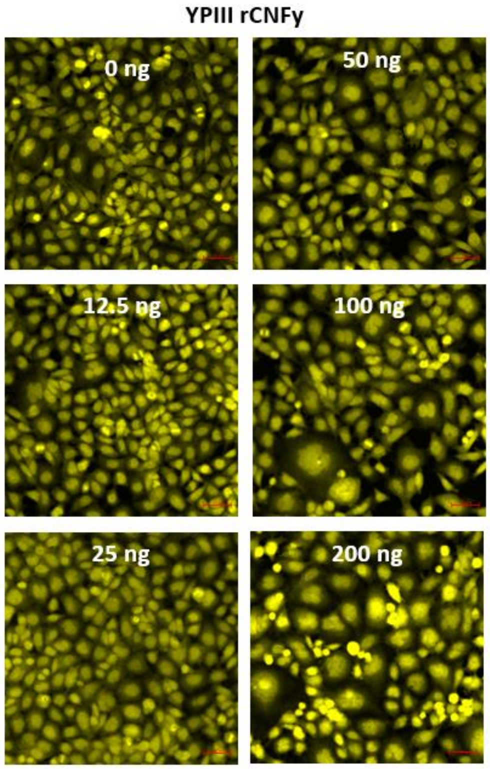

Figure S5. Multinucleation of HeLa cells after treatment with CNFy expressed and purified from *Y. pseudotuberculosis* YPIII. The HeLa cells were exposed to different concentrations of the purified toxin as described in the Materials and Methods section. To visualize the nuclei, they were stained with the nuclear stain DAPI (false color yellow). Scale Bar - 50µm.

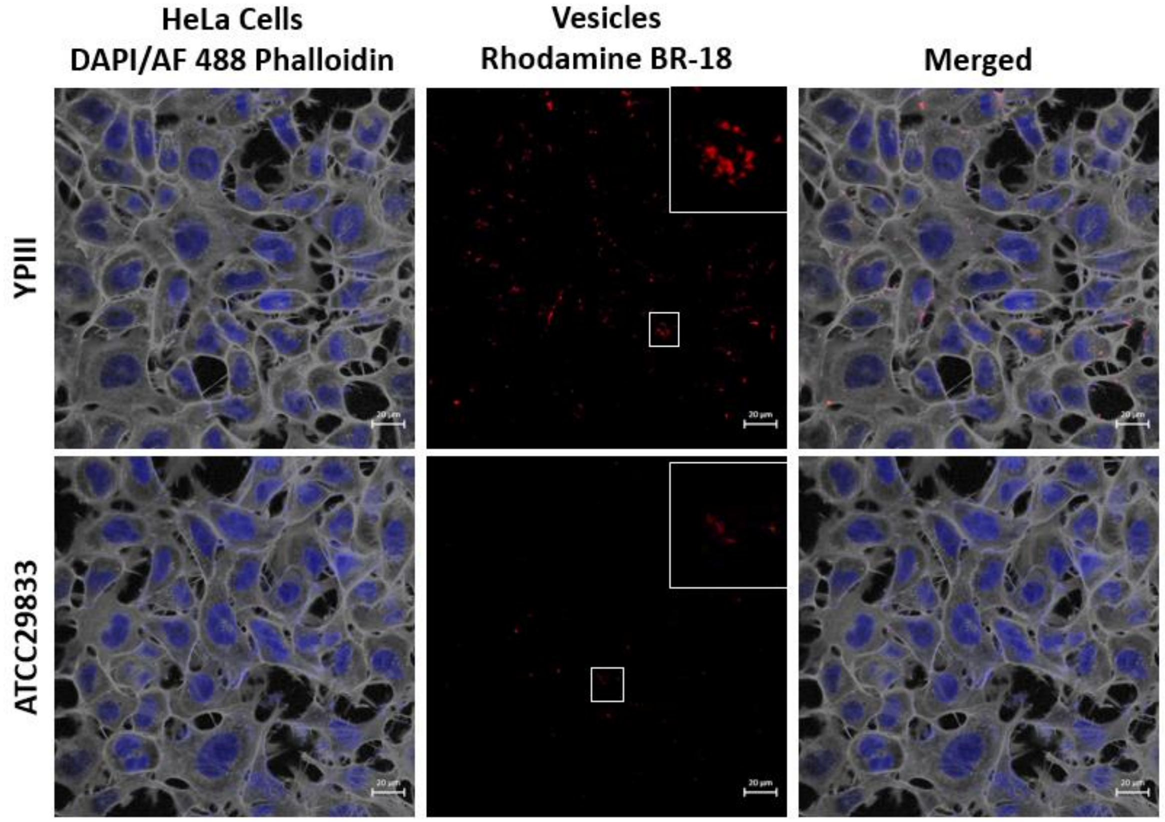

Figure S6. Image of MVs from *Y. pseudotuberculosis* YPIII or ATCC 29833 associated with HeLa cells. The MVs were labeled red using Octadecyl Rhodamine B Chloride (R18) while the actin cytoskeleton was stained with Alexa Fluor 488 phalloidin (represented in false color as grey) and the cellular DNA counter-stained blue using DAPI. The images were captured using confocal microscopy. The inset boxes show an expanded view of the MVs. Scale bar-20μm.

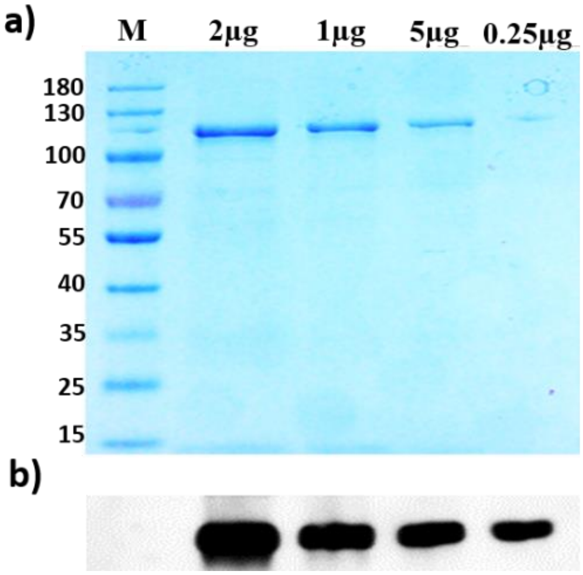

Figure S7. SDS-PAGE and western blot analyses of the CNFy expressed in *Y. pseudotuberculosis* YPIII. The CNFy protein was purified by affinity chromatography using Ni-NTA agarose beads. **a)** Different concentrations of CNFy sample (2 μg to 0.25 μg based on the Bradford assay) were seperated on a 10% SDS-PAGE gel and visulaized after staining with colloidal coomassie. **b)** Western blot analysis of the same protein concentrations using the Penta-His HRP conjugate Kit (Thermo-Fisher Scientific, USA). M - PageRuler Plus Prestained Protein Ladder.
